# A New Concept for Carotid Artery Stenting: Coating the Atherosclerotic Plaque by Covered Stent before Bare Stent Implantation

**DOI:** 10.1155/2016/3125629

**Published:** 2016-02-02

**Authors:** Erol Akgul

**Affiliations:** Radiology Department, Medical Faculty, Cukurova University, 01330 Adana, Turkey

## Abstract

In carotid artery stenting (CAS) procedures, distal embolism, periprocedural stent thrombosis, and 30-day stroke due to the plaque fragmentation and protrusion caused by stent implantation and balloon dilation are frequent complications. In this technical case report, a case is presented of extracranial carotid artery stenosis treated with a covered stent and subsequent implantation of a bare stent. In addition, the possibility is discussed that this new technique prevents the distal microembolic complications, periprocedural stent thrombosis, and 30-day stroke of extracranial CAS.

## 1. Introduction

In carotid artery stenting (CAS) procedures, distal embolism due to the plaque fragmentation caused by stent implantation and balloon dilation is the most frequent complication. Periprocedural stent thrombosis, 30-day stroke, and late in-stent stenosis are also possible complications causing serious neurologic problems. During stent implantation and/or balloon dilation before and after stenting, disruption of plaque and distal migration of the plaque particles may cause symptomatic or asymptomatic ischemic events seen on diffusion-weighted magnetic resonance imaging (MRI) even though protection devices are used. Many physicians may encounter plaque particles filling the filter and causing symptomatic cerebral embolism in some CAS cases, especially with ulcerated, fibrofatty plaque and severe stenosis [1, 2]. Additionally, after stent implantation and/or balloon dilation, plaque protrusion inside the stent may cause periprocedural stent thrombosis, 30-day stroke, and late in-stent stenosis [3, 4]. To reduce the plaque fragmentation and prevent plaque protrusion inside the vessel lumen, the CAS technique was changed in our clinic. In all CAS cases, we perform one of two new techniques. One is coating the atherosclerotic plaque with a covered stent before bare stent implantation; the other is double stenting with sequential balloon dilation. In both techniques, distal protection devices are used. In this technical case report, I present a case of extracranial carotid artery stenosis treated with a covered stent followed by implantation of a bare stent, and I discuss whether this new concept may prevent the distal microembolic complications, periprocedural stent thrombosis, and 30-day stroke of extracranial CAS.

## 2. Case Presentation and Technique 

**Figure 1:**
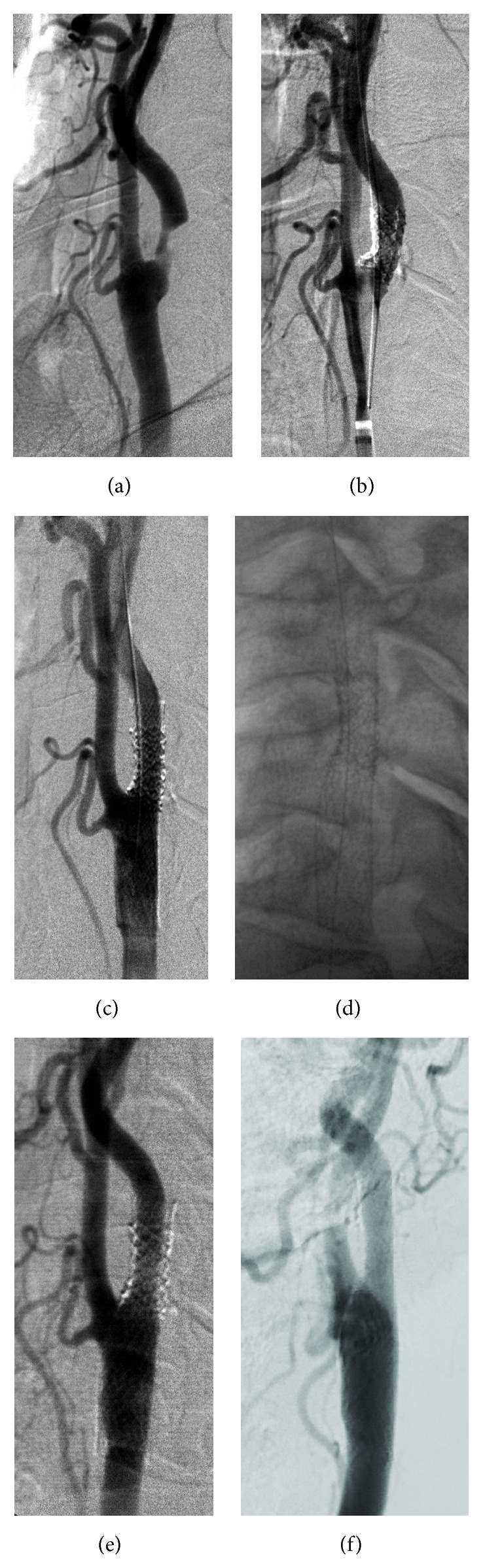
Digital subtraction angiogram shows eccentric, short segment plaque in the internal carotid artery next to the carotid bulb (a). After placing a protection filter (Emboshield NAV6), a covered stent (Direct Stent) was deployed to cover the plaque fully (b). In (c), angiogram shows no residual stenosis following both the insertion of a tapered, closed-cell stent (X-act) covering the covered stent and extending from the common to the internal carotid artery and the dilation of a 5 mm balloon. The stents are clearly visible in (d). Follow-up angiogram performed at the 6th (e) month shows no restenosis but minimal intimal hyperplasia inside the stents. The hyperplasia inside the stents is seen unchanged in the 12th-month angiogram (f).

A 59-year-old male patient suffering from transient ischemic attack (TIA) occurring two times in one year was admitted to our clinic for evaluation for CAS. The last TIA had occurred one week before admission. Doppler ultrasonography (US) and MR angiography (MRA) were performed and fibrofatty, eccentric, short plaque was seen causing stenosis over more than 70% of the proximal part of the left internal carotid artery (ICA). He had been on medicine for hypertension for about 10 years and had been smoking half a pack a day for about 40 years. Daily, 300 mg of acetylsalicylic acid (Aspirin; Bayer Healthcare, Germany) and 75 mg of clopidogrel (Plavix; Bristol-Myers Squibb/Sanofi Pharmaceuticals, NY, USA) were prescribed and the CAS was planned. Before the procedure, Aspirin and Plavix sensitivity were tested with VerifyNow (Accumetrics, San Diego, CA, USA). The patient was sensitive to Aspirin but resistant to Plavix. Ticlopidine (250 mg) (Ticlid, Sanofi-Aventis, Paris, France) was ordered two times a day and the procedure was performed two days later. Before the procedure, a diffusion-weighted MRI was performed to detect any new ischemic lesion caused by the CAS procedure. Diagnostic angiograms obtained under local anesthesia showed 75% stenosis consistent with the Doppler US and MRA (Figure 1). Heparin (5000 IU) was administered intravenously (IV) and then the left common carotid artery was catheterized with a 6-F, long shuttle sheath (Cook Inc., Bloomington, IN, USA) with the assistance of an exchange, hydrophilic 0.035-inch guide wire; a distal protection filter (Emboshield NAV6, Abbott, Redwood City, CA, USA) was placed inside the ICA, 4-5 cm away from the stenosis. A balloon-expandable stent graft (ePTFE polymer coated) 5 × 13 mm in size (Direct Stent-Stent Graft, InSitu Technologies®, Inc., Minnesota, USA) was deployed inside the proximal part of the ICA to cover and squeeze the plaque between the stent and the vessel wall, without covering the orifice of the external carotid artery. After performing angiography, a tapered, closed-cell carotid stent 9–7 × 30 mm in size (X-Act, Abbott, Redwood City, CA, USA) was implanted and a 5 × 20 mm monorail balloon was inflated inside the stents in the ICA part, both for proper apposition of the proximal and distal ends of the covered stent, for full opening of the stenosis without leaving any residue (Figures 1(a)–1(d)). During the covered stent implantation, the systolic blood pressure dropped from 185 to 90 mmHg and the pulse fell from 80 to 55/m. Atropine (0.5 mg) was administered IV urgently and at the end of the procedure the systolic blood pressure and pulse were 105 mmHg and 85/m, respectively. The filter was extracted and there was no debris inside it. The entrance site, the right common femoral artery, was closed with a 6-F closure device (Angio-Seal, St. Jude Medical, Minnesota, USA) for hemostasis. Physical examination showed no neurological deficit. A heparin IV (750 IU/h) was ordered for 24 h and a diffusion-weighted MR was performed one day later. There were no new ischemic lesions. The patient was discharged without any neurologic problems. Aspirin was recommended indefinitely and ticlopidine for 6 weeks. The patient was neurologically symptom-free on follow-up, and Doppler US performed in the 1st, 3rd, and 6th months and catheter angiography performed in the 6th and 12th months showed no in-stent stenosis but minimal intimal hyperplasia inside the stent (Figures 1(e) and 1(f)).

## 3. Discussion

In CAS, patients are still under high complication rates due to plaque disruption causing distal embolism and plaque protrusion resulting in periprocedural thrombosis, 30-day stroke, and late in-stent stenosis, in spite of the operator experiences and evolving technology that have decreased CAS complications. Current stent designs may trap larger fragments, but they may not efficiently prevent microemboli because gaps between the stent struts are too large. Furthermore, the introduction of proximal and distal protection devices has lowered the rate of periprocedural ischemic complications [5, 6]. Especially ulcerated, echolucent, fibrofatty vulnerable plaques and plaques causing severe stenosis are more prone to disruption and causing cerebral embolism during stenting and balloon dilation in spite of using protection devices. In some cases, it is possible that the disrupted plaque particles may fill the protection filter and cause symptomatic neurologic complications as well as asymptomatic brain lesions seen on diffusion-weighted MRI [7]. To reduce the distal embolism due to plaque particles, coating the plaque with a covered stent, in suitable vessels without tortuous anatomy compelling the insertion of the covered stent, will prevent fragmentation of the plaque as was done in this case. Therefore, fewer distal embolic lesions will be seen than in patients treated with a single stent.

Stroke seen from 24 hours to 30 days after the procedure is also a major complication in addition to periprocedural stroke [8]. This suggests that stroke occurring as a complication of CAS cannot be attributed solely to technical problems. Although the cause of late stroke after CAS is unknown, plaque protrusion may be one of the potential causes. The frequency of plaque protrusion into the stent after CAS was very low (4%) on angiography; Shinozaki et al. reported that the frequency of plaque protrusion into the stent was 7.8% by intravascular US in 77 consecutive CAS patients. They saw plaque protrusion in only 2 patients by angiography [9]. The most effective way to prevent plaque protrusion may be by using a covered stent. In suitable cases, plaque in the ICA and not extending to the common carotid artery can be jailed and squeezed between the graft and the vessel wall by a covered stent long enough to cover the plaque.

The major causes of late in-stent stenosis seen in CAS cases are the neointimal proliferation caused by self-expending stents, small postprocedural stent dimensions due to inefficient balloon dilation, and plaque protrusion inside the stent [10]. Plaque protrusion may also cause acute stent thrombosis and subacute in-stent stenosis, which is a devastating complication rarely seen in 0.04% to 2% of all cases [3]. The prevention of plaque protrusion inside the stent is important to decrease the rates of restenosis, acute thrombosis, and 30-day stroke. One way to prevent plaque protrusion is to cover the plaque with a stent graft as described in this technical report. The major and important limitation of this technique is the high rate of restenosis seen in patients treated with covered stents. In an aborted randomized trial, Schillinger et al. reported that a high rate of restenosis (38%, *n* = 3) was seen in 8 patients that had been treated with covered stents for carotid artery stenosis, while no restenosis was seen in their bare stent group [11]. The causes of the high rate of restenosis with stent grafts are unknown. The assumptions are that the buckling of the proximal and distal ends of the covered stent and the prevention of endothelization by the covered stent cause restenosis with excessive neointimal hyperplasia [6]. In this report, the technique is different and a bare stent is used to cover the covered stent and any remaining plaque beyond the covered stent. Inserting a bare stent inside the covered stent prevents the buckling of the distal and proximal ends of the covered stent. So it is hoped that the restenosis rate will be lower in our ongoing studies.

Another advantage of the covered stent is its usability without any distal protection devices in tortuous ICAs, in which a distal protection filter is impossible to place [5, 6].

In conclusion, some suitable cases of carotid artery stenosis have been treated in our clinic by coating the plaque with a covered stent (with a distal protection filter) before implantation of the bare stent. With this technique, the cerebral embolism due to plaque fragmentation and the 30-day stroke and stent thrombosis due to plaque protrusion can be reduced, but the high rate of restenosis in cases treated with covered stents may be still a problem.
